# The association between higher education and approximate number system acuity

**DOI:** 10.3389/fpsyg.2014.00462

**Published:** 2014-05-21

**Authors:** Marcus Lindskog, Anders Winman, Peter Juslin

**Affiliations:** Department of Psychology, Uppsala UniversityUppsala, Sweden

**Keywords:** approximate number system, arithmetic fluency, higher education, numeracy

## Abstract

Humans are equipped with an approximate number system (ANS) supporting non-symbolic numerosity representation. Studies indicate a relationship between ANS-precision (acuity) and math achievement. Whether the ANS is a prerequisite for learning mathematics or if mathematics education enhances the ANS remains an open question. We investigated the association between higher education and ANS acuity with university students majoring in subjects with varying amounts of mathematics (mathematics, business, and humanities), measured either early (First year) or late (Third year) in their studies. The results suggested a non-significant trend where students taking more mathematics had better ANS acuity and a significant improvement in ANS acuity as a function of study length that was mainly confined to the business students. The results provide partial support for the hypothesis that education in mathematics can enhance the ANS acuity.

## INTRODUCTION

Numbers are ubiquitous in modern society and from an early age, children in most western cultures learn to assign Arabic number labels to numerosities and to manipulate numbers using arithmetic rules. In addition to being able to learn formal mathematics humans are also believed to be equipped with an ability to represent numerical magnitudes without the use of symbols (e.g., Feigenson et al., 2004). This ability is common to human adults, infants and non-human animals and has even been documented in cultures lacking words for numerosities larger than five (Pica et al., 2004). The ability to represent numerosities non-symbolically is thought to be supported by a core cognitive system, the approximate number system (ANS), which represents magnitude in an approximate fashion with representations becoming more imprecise as numerosities increase (Dehaene, 2009; but see, Brannon et al., 2001). There are alternative accounts that reject the idea of an ANS altogether, instead proposing that numerosity judgments stem from multiple weighting of continuous visual cues (Gebuis and Reynvoet, 2012).

The precision, or acuity, of the ANS is often conceptualized as the smallest change in numerosity that can be reliably detected and has predominantly been quantified using a Weber fraction (*w*). ANS acuity is typically measured using paradigms where the participant is asked to identify which of two sets of objects is the more numerous, with too brief exposure for the two sets to be serially counted. Although the ANS is considered a primitive system that everyone can utilize, recent studies have documented substantial individual variability in ANS acuity (*w*:s for adults are often in the range 0.1–0.45, see; e.g., Pica et al., 2004; Halberda and Feigenson, 2008; Halberda et al., 2008; Tokita and Ishiguchi, 2012). The acuity improves with age from childhood to adulthood (Halberda and Feigenson, 2008; Halberda et al., 2012) but seems to peak at about age 30 followed by a decrease into old age.

Results from studies using brain imaging have indicated that the ANS has a neural basis in the intraparietal sulcus (IPS) on the lateral surface of the parietal lobe (Castelli et al., 2006) and studies on macaque monkeys (Nieder et al., 2002) have even identified specialized neurons (numerons) within the IPS that are selectively sensitive to specific numerosities [for a computational mechanism of numerosity estimation see Stoianov and Zorzi (2012)]. In addition to being activated by non-symbolic numerosities the IPS responds to numbers presented in different formats and modalities and when arithmetic tasks are performed (Piazza et al., 2004).

These findings suggest an association between the ANS and formal mathematics. Such correlations have recently been investigated and several studies with children have documented positive associations (e.g., Halberda et al., 2008; Inglis et al., 2011; Libertus et al., 2011; Mazzocco et al., 2011a,b). For example, Halberda et al. (2008) found that ANS acuity at age 14 correlated with previous math achievement, even after controlling for a set of cognitive abilities. While studies with children have repeatedly documented ANS-math correlations, results from studies on adults are somewhat more mixed (Gebuis and van der Smagt, 2011; Inglis et al., 2011; Castronovo and Göbel, 2012; Halberda et al., 2012; Lindskog et al., 2013a). Both methodological differences (Gebuis and van der Smagt, 2011; Lindskog et al., 2013a) and the possibility that the ANS-math relationship changes over age Inglis et al. (2011) has been suggested as possible explanations for the mixed results.

A relationship between ANS acuity and achievement in mathematics could indicate a causal link, but the direction of such a link is in dispute. An association between the precision of the ANS and mathematical achievement may exist because the neural correlates of the ANS lay the foundation for higher level arithmetical concept (Gilmore et al., 2007; Halberda et al., 2008). The observed correlations between ANS acuity and mathematical ability, both for children and for adults, together with the observation that the ANS is an evolutionary primitive system that can be found in several species (Feigenson et al., 2004) has been taken to support the idea of the ANS being a prerequisite for formal mathematical ability (e.g., Dehaene et al., 1998; Halberda et al., 2008). This causal direction is also supported by the fact that there exist stable individual differences in ANS acuity well ahead of the introduction of formal math education (Libertus and Brannon, 2010) and that developmental dyscalculia is associated with ANS impairment (Piazza et al., 2010; Mazzocco et al., 2011a). Also, an experimental study (Park and Brannon, 2013) demonstrated that training with non-symbolic addition and subtraction had an enhancing effect on math performance, in support for causality in this direction. Further, a recent study of Starr et al. (2013) found that preverbal number sense in 6-month-old infants correlated with math ability three years later and suggested that this indicates that number sense in infancy is a building block for later mathematical ability.

Another proposed possibility is that participating in math education will sharpen the ANS. Recent studies have investigated how differences in ANS acuity are related to a person’s level of education (Zebian and Ansari, 2011; Castronovo and Göbel, 2012; Nys et al., 2013; Piazza et al., 2013). The results are not conclusive, with some studies indicating that the level of education influences ANS acuity (Nys et al., 2013; Piazza et al., 2013). Nys et al. (2013) showed that unschooled adults had poorer ANS acuity than schooled adults with formal math education (but see Zebian and Ansari, 2011; Castronovo and Göbel, 2012). Effects of age and education are hard to separate in developed countries in which most individuals receive arithmetic education in childhood. However, Piazza et al. (2013) studied the Mundurucú in the Amazon among whom only some received schooling at adulthood. The results showed a significant positive effect of education (specific to numeracy instruction) on ANS acuity after controlling for chronological age. This study also reports that in Mundurucú, those not exposed to education stay at the same level as western children of age 6, about the age when they start to receive formal arithmetic education. This result suggests that ANS acuity is not fixed by genetic predispositions, but can be altered even in adulthood.

Most previous studies have investigated the impact of introductory level education whereas only one study (Castronovo and Göbel, 2012) has investigated the impact of higher education on ANS acuity. The authors of this study compared the ANS acuity and arithmetic ability between university psychology and mathematics students. While the study found a difference in arithmetical ability between the two groups there was no corresponding difference in ANS acuity and there was no correlation between ANS acuity and arithmetical ability. This study used a paradigm to measure ANS acuity where the non-symbolic stimuli are presented sequentially, one after the other, separated by a short inter stimulus interval. Previous research has indicated that this paradigm may suffer from low “predictive validity,” in contrast to paradigms where the stimuli are presented simultaneously. Lindskog et al. (2013a) showed with a within-subjects design that whereas ANS acuity obtained in a simultaneous presentation task predicted arithmetic fluency, acuity obtained with a sequential presentation failed to do so. Lindskog et al. (2013a) proposed that the sequential presentation may make attention or working memory abilities overshadow ANS precision as predictor of performance. Thus, it is possible that the lack of education effect on ANS in (Castronovo and Göbel, 2012) may have been a result of the chosen sequential paradigm.

### THE PRESENT STUDY

As described above, different views exist about a potential link between ANS and math performance. We will refer to the view that ANS plays an important role as a foundation when acquiring math due to the overlapping representations as the *Foundation hypothesis*. The opposite view, that it is the culturally learned symbolic skills that bring about an improvement in the non-symbolic system will be referred to as the *Cultural refinement hypothesis*. Although most previous research advocates one of the two possibilities over the other it is important to acknowledge that they are not necessarily mutually exclusive. Rather they might, for example, both be relevant but under different periods of human development and the relation might be bidirectional.

In the present study, we tried to distinguish between predictions of the above hypotheses by investigating the association between higher education and ANS acuity by measuring both ANS and arithmetic fluency in three student groups. We selected groups based on their major field of study (humanities, business, and mathematics) and the amount of mathematics education included in the specific field. Both first year and third year students were tested to evaluate a possible causal effect of higher education. In line with previous research we expected arithmetic fluency and numeracy to be highest in math students (see, e.g., Castronovo and Göbel, 2012) followed by business students and students of the humanities. This is probably at least partly because of self-selection effects. We expected those of low mathematic ability to be less inclined to seek math education and more inclined to follow education in the humanities, which almost entirely lacks mathematical content. We expected business students to be in-between the other groups, neither repelled by mathematical course content, nor actively in pursuit of this content *per se*.

Because of the previous findings discussed above (Lindskog et al., 2013a) we included measures from both types of stimulus presentation and expected a correlation with arithmetic fluency only for the simultaneous measure. This is because we hypothesize that other factors, such as working memory may play a more important role with sequentially presented stimuli. If ANS acuity is important for mathematical ability, we also expected it to be possible to predict performance in an arithmetic fluency task from ANS acuity even after controlling for general cognitive ability. This should, again, however, only be the case for the simultaneous measure of ANS acuity. To investigate if ANS acuity is primarily related to lower level arithmetical skills or more complex mathematical skills we also included a measure of numeracy.

It follows from the predicted rank ordering of students with respect to arithmetic fluency and the predicted relationship between ANS acuity and arithmetic fluency that we should expect a rank ordering of the groups with respect to ANS acuity for simultaneously presented stimuli, with best acuity in math students, followed by business students and students of the humanities. This effect is predicted by the foundation hypothesis, given the assumption that people with ANS acuity and thus high math ability choose disciplines that involve mathematics. This pattern may also be expected on account of the cultural refinement hypothesis, considering that (at least in the Swedish school system) those high in math ability, on average, are likely to have been exposed to more mathematical education prior to entering the university educations. Thus, we expected to find a main effect of student group. An effect of study length would support the proposed cultural refinement hypothesis. Such an effect should occur, however, only for business – and math students rather than for the humanities students, who receive virtually no mathematical training in their university education. Accordingly, the effect predicted by the cultural refinement hypothesis takes the form of an interaction between study length and discipline. The foundation hypothesis predicts that exposure to math in studies should have no effect and thus neither a main effect of study length nor an interaction between study length and study discipline. Because we do not expect a correlation between ANS acuity for sequentially presented stimuli and arithmetic fluency, we did not expect a corresponding rank ordering of student groups for sequentially presented stimuli.

## MATERIALS AND METHODS

We used a between subjects procedure with participants recruited from different undergraduate curricula; math, business and humanities. All participants took part in the following tasks described in detail below; In the ANS acuity tasks participants discriminated between which of two sets of dots (presented either simultaneously or in a temporal sequence) that was more numerous. The dependent measure was the estimated weber fractions (*w*). The RAPM task consisted of a short version of Raven’s progressive matrices. The arithmetic fluency task consisted of a timed test of mental arithmetic. The numeracy task was the adaptive Berlin Numeracy Test.

### ETHICS STATEMENT

All participants received an information sheet on the study and provided verbal informed consent before undertaking the study. The nature of the study was not in any way invasive, or unpleasant, did not involve deception, part taking was voluntary and participants were explicitly told that they could abort the study whenever they wished. In addition, no personal information was recorded in a way that could make identification of a specific participant possible. The research and the consent procedure were approved by the ethic committee of Uppsala University. There was no further documentation of the informed consent, and such documentation was not a requirement of the ethic committee.

### PARTICIPANTS

Participants (60 male, 40 female) were undergraduate students from Uppsala University with a mean age of 22.6 years (SD = 3 years). Participants were recruited from each of three student groups [humanities (*N* = 38), business (*N* = 31), mathematics (*N* = 31)] based on the amount of mathematics in their field of study. Participants for the *humanities* group were recruited from students majoring in philosophy, gender studies, history, art, literature or educational studies. The *business* students were recruited from students majoring in economics, political economics, business administration, or political science. Finally, *mathematics* students were recruited from students majoring in mathematics, physics, or engineering physics. Due to the heterogeneity of these groups we have no exact quantification of mathematical course content. Neither the humanities nor the business students receive explicit mathematical education. The humanities students receive virtually no mathematic content, whereas business students are confronted with math indirectly by applications and practical exercises relating to economics. Math students do receive approximately 600 h of explicit mathematical education per term. Participants were either first year students (*N* = 51) or third year students (*N* = 49), in their specific field of study. They received a movie ticket for their participation.

### MATERIALS AND PROCEDURE

Participants carried out a set of four tasks, described in detail below, developed to measure ANS acuity, intelligence, and arithmetic fluency.

#### ANS acuity task

The ANS acuity task was based on Lindskog et al. (2013a). On each of the 300 trials, participants saw spatially intermixed blue and yellow dots on a monitor. Exposure time (300 ms) was too short for the dots to be serially counted. Half of the trials had blue and half had yellow as the more numerous set on a gray background. The dots varied randomly in size, subtending visual angles varying approximately between 0.5° and 1°. The stimuli were presented as a single large array in the center of the screen on an area subtending a visual angle of approximately 17°. The dots were arranged in a non-overlapping (i.e., each dot was spatially distinct from each other dot) fashion, similar to the “intermixed” condition of (Price et al., 2012)^1^. The stimuli in the sequential presentation were thus identical to those in the simultaneous condition in every possible respect but for a temporal separation of the two dot sets.

The participants judged which set was more numerous by pressing a color-coded keyboard button. ANS threshold for obtaining 80% correct discriminations was estimated by an adaptive method, the ZEST algorithm (King-Smith et al., 1994). The algorithm calculates the stimulus difference for each trial based on the performance on earlier trials in the discrimination task. The ZEST algorithm uses all responses in previous trials for optimal estimation of the difference between stimuli presented in the next trial and converges to the threshold estimate, *w*, for achieving the desired percentage of correct responses^2^. After each trial a probability density function (a prior PDF) of *w* is multiplied with a likelihood function of obtaining the response (correct or incorrect). The result is an updated density function (a posterior PDF). The mean of the updated PDF is used to determine *w* for the next trial. A loop was used that searched for the nearest *w* ratio with integer composition of dots with the constraint that the total number of dots not exceeded 33, and a minimum of six dots of the less numerous set. The larger numerosity varied between 7 and 25 in number (*M* = 12.8, SD = 3.3, Md = 13). The smaller numerosity varied between 6 and 16 (*M* = 10.2, SD = 2.6, Md = 10). The mean weber fraction of all stimuli was 0.258 (Min = 0.062, Max = 2.14. SD = 0.12). If several ratios were found, the ratio with lowest total number of dots was used. The initial PDF was a normal distribution of *w* with a mean of 0.23 and a standard deviation of 0.58. The initial estimate was based on the median *w* for a large number of participants (≈200) tested in our lab^3^. This estimate is also consistent with estimates documented in previous research (Pica et al., 2004; Halberda and Feigenson, 2008; Halberda et al., 2008; Tokita and Ishiguchi, 2012). We used both a simultaneous and a sequential version. In the sequential version, the two numerosities were separated by an ISI of 300 ms with a blank screen. The simultaneous and sequential tasks were presented in two separate blocks with all participants completing the simultaneous task first^4^. For both tasks the algorithm simultaneously estimated two *w* values based on randomly ordered intermixed trials (150 trials each). The same initial normal PDF (μ = 0.23, σ = 0.58) was used for both *w* estimates in both tasks.

#### Raven’s matrices

Participants completed a subset of Raven’s progressive matrices (Raven et al., 1998) based on (Stanovich and West, 1998; see also, Carpenter et al., 1990). Participants were first instructed on the task. They were then allowed two of the 12 test items before completing 18 of the test items (item 13 through 30) with a 15 min time limit. Participants were instructed to try to complete all 18 items within the time limit.

#### Arithmetic fluency

The arithmetic fluency task was based on (Gebuis and van der Smagt, 2011) and consisted of four sets of arithmetic problems; addition, subtraction, multiplication and division. Participants had 150 s to complete as many problems as possible for each set. In each set, problems increased in difficulty by addition of digits and requiring borrowing or carrying. For example the first three problems in the addition and multiplication sets were 2 + 7, 12 + 9, and 38 + 17, and 2 × 3, 3 × 6, and 4 × 7 respectively. The order of sets was counterbalanced over participants.

#### Numeracy

Participants completed a Swedish version of the adaptive Berlin Numeracy Test (Cokely et al., 2012). The test measures participants’ level of numeracy. That is, their understanding of, and ability to process, numerical concepts, particularly their ability to comprehend risk and transform probabilities (Lipkus et al., 2001; Peters et al., 2006; Reyna et al., 2009). Thus, numeracy is an ability that requires mathematical skills over and above those needed to perform well in the arithmetic fluency task. The adaptive Berlin Numeracy Test is a short test that measures statistical numeracy and risk literacy in a psychometrically sound way (Cokely et al., 2012). It consists of four questions in an adaptive structure where participants answer 2–3 questions depending on their performance. The adaptive structure adjusts the difficulty of subsequent questions based on the prior performance of the participant and is constructed to make all questions have about a 50% probability of being answered correctly. The test assigns participants to one of four skill-levels (1–4) of numeracy. The first question, which is intended to give an approximate median split for a student population, reads: “Out of 1,000 people in a small town 500 are members of a choir. Out of these 500 members in the choir 100 are men. Out of the 500 inhabitants that are not in the choir 300 are men. What is the probability that a randomly drawn man is a member of the choir?” (Cokely et al., 2012).

## RESULTS

All measures were scanned for outliers separately, and any data points deviating more than three standard deviations (|*z*| > 3) from the mean performance in each measure were removed from subsequent analyses. This procedure resulted in less than 1% of excluded data points^5^. The arithmetic fluency task included four subtasks, one for each of the four basic rules of arithmetic. We used the number of correct answers in each of these subtasks as dependent measures of arithmetic fluency. In addition, we calculated two composite measures. The first was the sum of correct answers over all four tasks measuring the overall arithmetic fluency (OAF). We also used a measure of a more basic arithmetic fluency, calculated as the sum of correct answers in the addition and subtraction tasks (addition/subtraction fluency: ASF)^6^. There were no age [*w*_sim_: *r*(93) = -0.05, *p* = 0.66; *w*_seq_: *r*(93) = -0.09, *p* = 0.40] or gender [*w*_sim_: *t*(95) = 1.03, *p* = 0.31; *w*_seq_: *t*(93) = 1.16, *p* = 0.25] effects on either measure of ANS.

**FIGURE 1:**
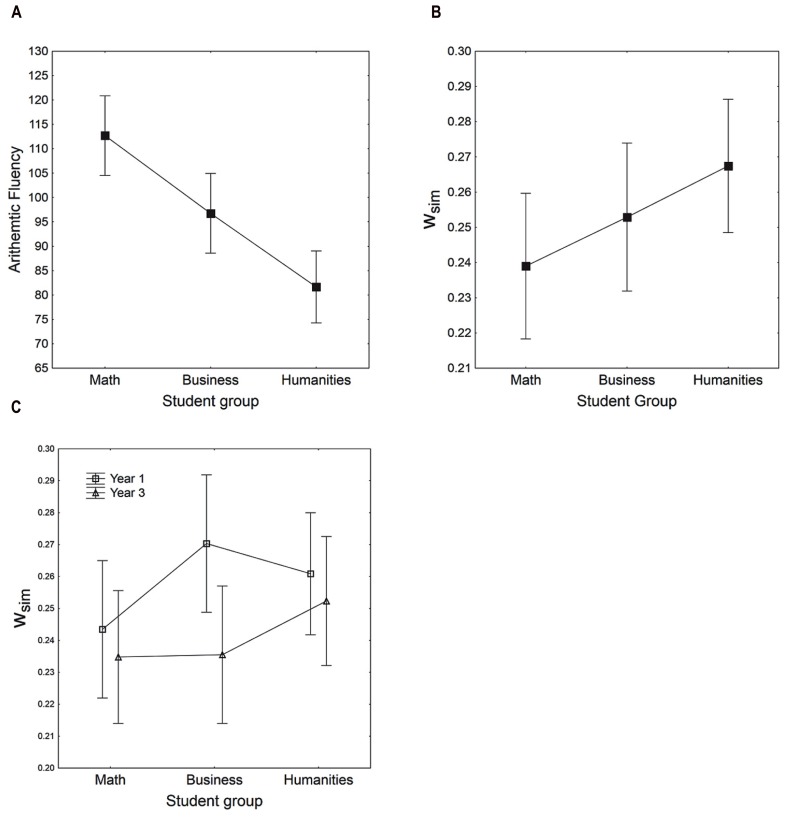
**(A)** Overall arithmetic fluency (OAF) as a function of student group. **(B)** Simultaneous ANS acuity *w*_sim_ as a function of student group. **(C)** Simultaneous ANS acuity *w*_sim_ as a function of student group and year of study (Year 1 or Year 3). The y-axes do not start from 0. Vertical bars denote 95% confidence intervals.

In the statistical analysis, and to maximize the statistical power, we followed the standard practice of testing predictions that are strongly theoretically motivated by *a priori* contrasts (see, e.g., Howell, 1997, pp. 350–351), where other main and interaction effects are tested by standard a posteriori tests to allow detection also of possible unpredicted effects. We performed a two-way ANOVA on OAF with study discipline and study length as the independent variables. An *a priori* contrast for the predicted monotonically decreasing trend for OAF across the student groups (mathematics, business, humanities) was statistically significant [*F*(1,93) = 31.2, *p* < 0.001, *partial* η^2^ = 0.252]. As illustrated in **Figure 1A**, there was a large decreasing trend in the predicted direction. The a posteriori omnibus test for the main effect of study length was not significant [*F*(1,93) = 1.427, *p* = 0.235, *partial* η^2^ = 0.015], but the omnibus test for the interaction was significant [*F*(2,93) = 3.5, *p* = 0.033, *partial* η^2^ = 0.071]. The interaction amounts to the “slope” depicted in **Figure 1A** being somewhat steeper at Year 3 than Year 1, which is due to an increase in arithmetic performance in business and math students, but not in humanities students.

We performed a two-way ANOVA on simultaneous ANS acuity *w*_sim_ with study discipline and study length as the independent variables. An *a priori* contrast for the predicted monotonically increasing trend for *w*_sim_ across the three student groups (mathematics, business, humanities) was marginally significant [*F*(1,91) = 2.9, *p* = 0.09, *partial* η^2^ = 0.031; see **Figure 1B**]. In regard to study length the prediction of a cultural refinement hypothesis was that if math education improves the ANS acuity, it should improve among the math and the business students, but not among the students in the humanities (i.e., which receive no education in mathematics). An *a priori* contrast for this predicted interaction for *w*_sim_ is statistically significant [*F*(1,91) = 4.374, *p* = 0.039, *partial* η^2^ = 0.021]. The effect size is small, and as illustrated in **Figure 1C**, it is qualitatively different from what is expected by the cultural refinement hypothesis in that the main improvement is with the business students. Math students had good ANS acuity already at Year 1, while, as expected, there was no improvement for students in the humanities. In the overall arithmetic test, first year business students performed more at level with humanities students than with math students. Third year business students, however, showed a performance closer to math students. The trend in *w*_sim_ across the three student groups failed to reach statistical significance by conventional criteria (*p* = 0.09). This could be because of poor statistical power due to the heterogeneity in the independent variable of interest (arithmetic performance) in the business students as well as to our limited sample size. To test the student group effect with more statistical power, we accordingly pooled the groups with best performance (math students and third year business students) into a high performance group (*M* = 109.0 solved problems) and those with poorest performance (humanities students and first year business students) into a low performance group (*M* = 84.8 solved problems). This division resulted in two groups where the groups performing on average below the overall median (98.5) were collapsed into one group, and the groups performing above the overall the median were collapsed into the other group. In this way, we receive an increase in sample size and more clear-cut differences in student groups with respect of the independent variable. An independent *t*-test showed that *w*_sim_ was significantly worse [*t*(95) = 2.7, *p* < 0.01] in the low performance group (*M* = 0.260), than in the high performance group (*M* = 0.237). In addition, the standard omnibus test for the main effects of study length was statistically significant [*F*(1,91) = 4.1, *p* = 0.045, *partial* η^2^ = 0.043], where third year students on average had better ANS-precision (*M* = 0.24, SEM = 0.006) than first year students (*M* = 0.26, SEM = 0.006). Again, as illustrated in **Figure 1C**, this significant main effect is secondary and driven by the significant interaction, where in practice most of the improvement in the ANS acuity as a function of study length is confined to the business students.

For a corresponding analysis of sequential ANS acuity *w*_seq_ there were no significant main effects or significant interaction (smallest *p* = 0.30). As predicted we do not observe any rank order across student groups with this measure.

An effect of study length could mean a causal effect of studies on the ANS. An alternative possibility is that this is an effect of selective attrition in that less study apt individuals will “drop out” between the first and the third year because of failure to reach the course requirements. To control for this possibility, we performed analyses separately for each student group while controlling for study aptitude^6^. ANCOVA analyses, with *w*_sim_ as dependent variable, study length as independent variable and RAPM as covariate showed that the effect is significant in the business student group [*F*(1,27) = 6.2, *p* = 0.018. adjusted means: first year 0.27, third year 0.23], but not in the other two groups [humanities: *F*(1,33) = 0.33, *p* = 0.63, math: *F*(1,28) = 0.01, *p* = 0.92]. In sum: for simultaneous ANS acuity (*w*_sim_) there was a trend in the predicted direction across the student groups, and a significant effect of study length that was mainly confined to business students.

We investigated the relationships between ANS acuity, arithmetic fluency, numeracy, and intelligence by calculating correlations for each ANS-measure. Arithmetic fluency is presented separately for the four arithmetic operations, OAF, and ASF. The results, summarized in **Table 1**, indicate that even though there is a significant relationship between the two ANS acuity measures [*r*(94) = 0.22, *p* = 0.03] only the simultaneous measure is significantly associated with the measures of arithmetic fluency, intelligence and numeracy. All correlations for the sequential measure are very close to 0, and in all cases except numeracy, significantly lower than the corresponding correlations for the simultaneous measure.

**Table 1:** Pairwise correlations (Pearson correlation) between the simultaneous (*w*_**sim**_) and sequential ANS measure (*w*_**seq**_) and performance on addition (Add), subtraction (Sub), multiplication (Mult), addition/subtraction fluency (ASF-addition and subtraction combined), division (Div), overall arithmetic fluency (OAF), Raven’s matrices (RAPM), and Berlin Numeracy Test (numeracy).

Arithmetic fluency									
ANS task	Add^a^	Sub^a^	Mult^a^	Div^a^	ASF^a^	OAF^a^	RAPM^a^	Numeracy	*w*_*sim*_
*w*_sim_	-0.35^**^	-0.32^*^	-0.41^***^	-0.33^**^	-0.36^**^	-0.40^***^	-0.36^**^	-0.29^*^	–
*w*_seq_	-0.01	0.04	0.03	0.04	0.01	0.03	-0.08	-0.19	0.22^*^

a Indicates a significant difference between correlation strengths for the two ANS measures (*p* < 0.05). *p < 0.05, **p < 0.001, ***p < 0.0001.

**FIGURE 2:**
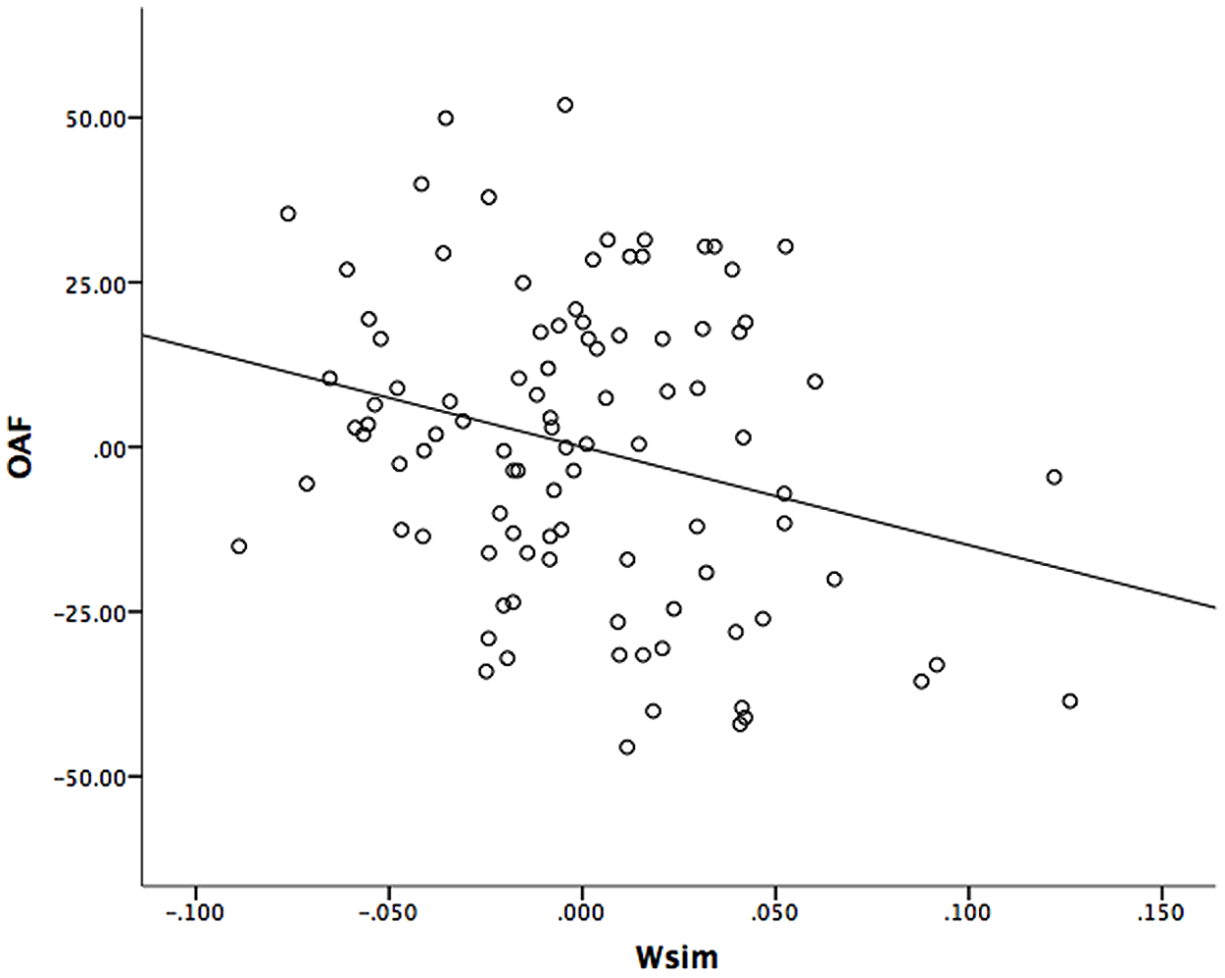
**Partial regression plot of OAF against *w*_sim_.** The plot illustrates the partial correlation between OAF and *w*_sim_ after the linear effects of Ravens’ is removed.

The results in **Table 1** suggest that it is possible to predict performance in mathematical abilities (OAF and numeracy) from ANS acuity measured with a simultaneous but not with a sequential task. However, the strong correlation between RAPM and *w*_sim_ implies that the predictive power of *w*_sim_ might disappear after controlling statistically for the effects of intelligence. We conducted four separate hierarchical multiple regression analyses, one for each measure of mathematical ability (OAF and numeracy) with RAPM entered as predictor in the first step and *w*_sim_ or* w*_seq_ entered as single predictor in the second step^8^. The results, summarized in **Table 2**, show that including *w*_sim_ adds predictive value over and above the effect of intelligence by a significant increase in explained variance from step one to step two. That is, *w*_sim_ is a significant predictor of OAF even after controlling for the effect of intelligence. However, *w*_sim_ does not contribute significantly over RAPM to predict numeracy. **Figure 2** illustrates the unique contribution of *w*_sim_ to OAF by plotting the partial correlation between OAF and *w*_sim_ after the linear effect of Ravens’ is removed. The results also show that the sequential ANS acuity measure cannot significantly predict any of the two math abilities.

**Table 2:** Hierarchical multiple regression models with overall arithmetic fluency (OAF) and Berlin Numeracy Test (numeracy) as dependent variables and Raven’s matrices (RAPM), Step 1, and simultaneous (*w*_**sim**_) or sequential (*w*_**seq**_) ANS measures, Step 2, as predictors. Change in *R*^**2**^ (Δ*R*^**2**^), beta weights (β), and total *R*^**2**^ are reported for the respective models.

Dependent variable				
	OAF		Numeracy	
Predictor	Δ*R*^2^	β	Δ*R*^2^	β
Step 1 RAPM	0.21^**^	0.46^**^	0.15^**^	0.39^**^
Step 2 *w*_sim_	0.06^*^	-0.25^*^	0.02	-0.13
Step 2 *w*_seq_	0.006	0.08	0.02	-0.15
Total *R*^2^ (*w*_sim_)	0.27^**^		0.16^**^	
Total *R*^2^ (*w*_seq_)	0.22^**^		0.18^**^	
*N*	97		97	

* *p* < 0.05, **p < 0.001.

## DISCUSSION

It has been proposed that humans are equipped with a core cognitive system, the ANS, which supports the representation of non-symbolic numerosities. Previous research has revealed associations between the precision with which the ANS can represent such numerosities and achievement in mathematics (e.g., Halberda and Feigenson, 2008; Halberda et al., 2008; Libertus et al., 2012). However, the question of whether ANS acuity lays the foundation for math achievement or if math education sharpens the ANS is unresolved. While some studies give support to the former conclusion (Halberda and Feigenson, 2008; Libertus et al., 2012) others indicate the opposite causal pathway (Nys et al., 2013; Piazza et al., 2013). The present study contributes to this debate by investigating how higher education with varying amounts of mathematics education influences ANS acuity. Results gave partial support for the cultural refinement hypothesis in that third year business students had better ANS acuity than first year business students, when controlling for study aptitude. This evidence is obviously correlational in nature, but it suggests the presence of a causal link in which being exposed to formal math education has an effect of improving ANS. However, we saw no effect of study length in the math students that would have been expected on the account of the cultural refinement hypothesis. This lack of effect for math students could possibly be a ceiling effect in that performance may only be improved up to a certain level. Thus, business students became as good as math students at non-symbolic discrimination with three years of math exposure, but there was no room for improvement in the math students. It is also possible that the effect depends on a more concrete form of mathematics including a large portion of arithmetic, rather than the more abstract mathematics taught to math majors.

The results revealed a predicted order of student groups for both arithmetic fluency and ANS acuity for simultaneously presented stimuli. Students majoring in mathematics performed best, followed by business students and students of humanities. The group differences in arithmetic fluency confirmed that self-selection effects may occur in study choice, which maybe is not surprising.

The differences in ANS between the groups were small and the contrast in the predicted order was only marginally significant. With a test maximizing the actual group differences in arithmetic fluency, the corresponding differences appeared more clearly. The simultaneous ANS acuity measure predicted arithmetic fluency, even after controlling for intelligence, but failed to predict higher level mathematics in terms of numeracy. While the simultaneous ANS acuity measure was significantly correlated to all measures of arithmetic fluency, no such correlations were found for the sequential measure. Accordingly, there was no corresponding rank order of student groups for ANS acuity for sequentially presented stimuli. This was predicted on the basis of previous results (Lindskog et al., 2013a). However, the reasons for the lack of correlation with simultaneous presentation are unclear. It is important to remember that this difference cannot be attributed to a low reliability of the sequential measure, since both previous research (Lindskog et al., 2013a) and the present study indicates that the sequential measure is actually more reliable than the simultaneous measure. We speculate that the reason for this is that the sequential presentation may make other task demands, by requiring participants to hold information briefly in memory, make other individual characteristics, such as short term memory determine the performance. It is thus possible that short term memory suppresses the correlation with arithmetic fluency with sequential stimulus presentation. Accordingly, future work should use measures of short term memory and relate its role to sequential and parallel stimulus presentation. This is especially interesting because in previous work we have observed strong correlations between short term memory and arithmetic fluency. It should maybe also be pointed out in this connection, that performance in the sequential task is superior to performance with parallel stimulus presentation, so it is not the case that the correlation with arithmetic fluency breaks down because performance deteriorates.

Recent studies (Fuhs and McNeil, 2013; Gilmore et al., 2013; Szücs et al., 2013) suggest that the method of controlling for visual parameters used in some ANS discrimination task may affect results. These studies not only use size controlled vs. area controlled trials, but rely on “congruent trials” in which the more numerous array has larger dots and “incongruent trials,” in which this arrangement is reversed [see, e.g., Gilmore et al. (2013) for a discussion of the congruent/incongruent distinction]. As Szücs et al. (2013) concluded, this setup virtually turns the numerosity task into a Stroop task. Accordingly, Gilmore et al. (2013) showed that with this stimulus setting, for children, the task can be considered a measure of inhibitory control, rather than of ANS acuity. When controlling for inhibitory control, the correlation between ANS acuity and math performance disappeared. The authors suggested that inhibitory control can be important even for adults. Cappelletti et al. (2014) proposed that the elderly’s poorer numerosity judgments may be due to their deteriorated inhibitory processes. The current adaptive ANS test, however, does not control for perceptual variables. In a previous study (Lindskog et al., 2013a) we directly compared a measure controlling for perceptual variables with the present task and found similar correlations for both tests with arithmetic ability. Because this task has no inhibitory control demands, these results suggest that for young adults inhibitory control does not play a role in accounting for the relationship with arithmetic performance. However, future research should address this issue. Regarding this issue, we have previously argued (Lindskog et al., 2013b) against the use of incongruent/congruent trials. We find it plausible that this method can probably induce behavior in participants that they would not exhibit with stimuli that does not highlight perceptual variables. Indeed this method can even communicate a demand characteristic in that the fact that the psychologist has set things up so that dot size conspicuously covaries with numerosity implies that the participant is expected to respond to dot size. With the commonly used method of controlling for area on half of the trials, and size on the other half of the trials, rather than using stimuli for which the numerous set for some stimuli consists of larger dots, there are no “congruent trials,” and perceptual variables are probably much less psychologically salient.

The finding of a dissociation between simultaneous/sequential stimulus presentation and the correlation with ANS acuity, indicates that the way that stimuli are presented may play an important role. As has been shown in previous research (e.g., Halberda and Feigenson, 2008) our results replicate the finding (Lindskog et al., 2013a) that it is possible to predict math achievement from ANS acuity, even after controlling for general cognitive ability. However, we extend these previous findings by showing that while it is possible to predict the specific skill of arithmetic fluency, this does not extend to a more general math achievement in terms of numeracy. It should be an important issue for future research to address exactly which kind of mathematical skills, and at what degree of complexity, that is actually associated with ANS acuity by use of tests of different types of math abilities. The observation that business students improved in ANS acuity from the first to the third year of study suggests the possibility of a causal link from higher level education to non-symbolic discrimination, although clearly more evidence (preferentially experimental) is needed before this conclusion can be drawn. Due to the cross-sectional design of the study it is of course possible that the difference seen for the business students could be a result of attrition, with generally lower performing students (maybe with low ANS acuity) dropping out between the first and third year of study. The fact that controlling for RAPM in the business students did not eliminate the effect, can not, of course rule out the possibility that this is still an effect of attrition due to factors that are not captured by the rather crude RAPM measure, as a proxy for study aptitude.

What could the psychological mechanisms behind an effect of math schooling on the ANS be? A number of researchers have proposed tentative possible mechanisms. Castronovo and Göbel (2012) suggested the possibility that the acquisition of a symbolic, exact number system (ENS) involves profound changes in the cerebral network responsible for numerical processing. A progression, according to this view, would take place from predominance of the right IPS to involvement of both left and right IPS for symbolic and non-symbolic number processing with increasing age. The result would thus be a refinement of ANS into a second ENS, with an exact code for numbers within the language dominant left hemisphere. Nys et al. (2013) proposed two possible mechanisms. With exposure to symbolic numbers people can learn to attend more to numeric properties rather than perceptual variables, and to give the former properties more weight. It is also possible that there is a transformation in the way we process number so that the utilization of number could refine the underlying representation, increasing the distinctiveness of the underlying magnitude representations. Noël and Rousselle (2011) argued that in the same way as acquisition of color names may affect color perception, acquisition of number words in an ENS can enhance the ANS by sharpening boundaries between numerosities at the non-symbolic level. According to this hypothesis, they proposed that the reduced acuity of the ANS found in dyscalculics is a consequence rather than a cause of a dysfunction in the representation of symbolic numbers. Verguts and Fias (2004) showed that numerons in neural network fed with symbolic input can benefit from this with increased representational effectiveness, suggesting a link between primitive and higher order numeric abilities. The system originally devoted to non-symbolic discrimination after symbolic training could represent the same kind of information with more precision (see also, Stoianov et al., 2004).

It is an interesting venue for future research to investigate the influence of higher education on ANS acuity with longitudinal designs. Another apparent future research agenda in search for causal explanations is to demonstrate with direct manipulations that arithmetic exposure enhances non-symbolic discrimination.

## Conflict of Interest Statement

The authors declare that the research was conducted in the absence of any commercial or financial relationships that could be construed as a potential conflict of interest.

## Footnotes

1 This adaptive test does not control for perceptual variables such as cumulative area. However, we have previously evaluated the psychometric qualities of the test (Lindskog et al., 2013a) and showed that performance correlates strongly [*r* (adjusted for attenuation) = 0.88] with a commonly used test procedure (Halberda et al., 2008) in which area is controlled for on half the trials and dot size on the other half of trials. For a large number of participants, we have failed to observe any influence of either average dot size or cumulative area on performance that suggest that participants rely on these variables.

2 In contrast to most previous work (Barth et al., 2006; Halberda et al., 2008) we do not model *w* on the proportion of correct answers for a fixed set of stimulus difficulties. Rather, *w* is used as a measure of the stimulus difficulty for obtaining 80% correct discriminations. In addition, because our method does not use fixed stimulus difficulties it is not possible to calculate some of the additional measures [e.g., the numeric ratio effect or the numeric distance effect (Price et al., 2012)] used in previous research.

3 Using previously collected data to inform the mean of the initial PDF will help the ZEST algorithm to start in a plausible region and will thus make the procedure more efficient than if an arbitrary initial estimate was used. This choice should, however, not affect the final estimate of *w*. The median rather than the mean of the *w-*distribution was used because the observed *w-*distribution has a slight positive skew.

4 With the main interest being in correlations between individual characteristics we wanted all other sources of variance be as low as possible and therefore did not counterbalance task order. This could leave room for unwanted task order effects. In our previous studies (Lindskog et al., 2013a,b) we have found that participants are remarkably resilient to either practice or fatigue effects and therefore have little reason to believe that such order effects play any role in these data.

5 Due to technical problems one participant did not complete the sequential ANS-task and one participant completed neither of the two ANS-tasks.

6 The distinction between ASF and OAF is motivated both by findings in previous research and by theoretical considerations. First, previous research (Lindskog et al., 2013a) has indicated that ANS acuity is related to ASF. OAF includes two additional subtasks, division and multiplication, and previous research (Lindskog et al., 2013a) has shown that division is not related to ANS acuity and multiplication is related but to a much smaller extent than ASF. Further, addition and subtraction are often the first operations that children learn and include explicit calculations, also when performed by adults. In contrast, multiplication and division are operations that are learnt much later and often, at least with the magnitude of numbers used here, involve root learning. That is, an adult would seldom calculate 9 × 7 explicitly but rather retrieve the answer (63) from long term memory.

8 We conducted the same hierarchical regression analysis with ASF as dependent variable. This analysis revealed results highly similar to those found with OAF as dependent variable.

## References

[B1] Barth H. La Mont K. Lipton J. Dehaene S. Kanwisher N. Spelke E. (2006). Non-symbolic arithmetic in adults and young children. *Cognition* 98 199–222 10.1016/j.cognition.2004.09.011 15876429

[B2] Brannon E. M. Wusthoff C. J. Gallistel C. R. Gibbon J. (2001). Numerical subtraction in the pigeon: evidence for a linear subjective number scale. *Psychol. Sci.* 12 238–243 10.1111/1467-9280.00342 11437307

[B3] Cappelletti M. Didino D. Stoianov I. Zorzi M. (2014). Number skills are maintained in healthy ageing. *Cogn. Psychol.* 69C 25–45 10.1016/j.cogpsych.2013.11.004 24423632

[B4] Carpenter P. A. Just M. A. Shell P. (1990). What one intelligence test measures: a theoretical account of the processing in the Raven progressive matrices test. *Psychol. Rev.* 97 404–431 10.1037/0033-295X.97.3.404 2381998

[B5] Castelli F. Glaser D. E. Butterworth B. (2006). Discrete and analogue quantity processing in the parietal lobe: a functional MRI study. *Proc. Natl. Acad. Sci. U.S.A.* 103 4693–4698 10.1073/pnas.0600444103 16537401 PMC1450233

[B6] Castronovo J Göbel S. M. (2012). Impact of high mathematics education on the number sense. *PLoS ONE* 7:e33832 10.1371/journal.pone.0033832 PMC3338810 22558077

[B7] Cokely E. T. Galesic M. Schulz E. Ghazal S. Garcia-Retamero R. (2012). Measuring risk literacy: the Berlin Numeracy Test. *Judgm. Decis. Mak.* 7 25–47

[B8] Dehaene S. (2009). Origins of mathematical intuitions. *Ann. N. Y. Acad. Sci.* 1156 232–259 10.1111/j.1749-6632.2009.04469.x 19338511

[B9] Dehaene S. Dehaene-Lambertz G. Cohen L. (1998). Abstract representations of numbers in the animal and human brain. *Trends Neurosci.* 21 355–361 10.1016/S0166-2236(98)01263-6 9720604

[B10] Feigenson L. Dehaene S. Spelke E. (2004). Core systems of number. *Trends Cogn. Sci.* 8 307–314 10.1016/j.tics.2004.05.002 15242690

[B11] Frey M. C. Detterman D. K. (2004). Scholastic assessment or g? The relationship between the scholastic assessment test and general cognitive ability. *Psychol. Sci.* 15 373–378 10.1111/j.0956-7976.2004.00687.x 15147489

[B12] Fuhs M. W. McNeil N. M. (2013). ANS acuity and mathematics ability in preschoolers from low-income homes: contributions of inhibitory control. *Dev. Sci.* 16 136–148 10.1111/desc.12013 23278935

[B13] Gebuis T. Reynvoet B. (2012). The interplay between nonsymbolic number and its continuous visual properties. *J. Exp. Psychol. Gen.* 141 642–648 10.1037/a0026218 22082115

[B14] Gebuis T van der Smagt M. J. (2011). False approximations of the approximate number system? *PLoS ONE* 6:e25405 10.1371/journal.pone.0025405 PMC3192045 22022390

[B15] Gilmore C. Attridge N. Clayton S. Cragg L. Johnson S. Marlow N. (2013). Individual differences in inhibitory control, not non-verbal number acuity, correlate with mathematics achievement. *PLoS ONE* 8:e67374 10.1371/journal.pone.0067374 PMC3681957 23785521

[B16] Gilmore C. K. McCarthy S. E. Spelke E. S. (2007). Symbolic arithmetic knowledge without instruction. *Nature* 447 589–591 10.1038/nature05850 17538620

[B17] Halberda J. Feigenson L. (2008). Developmental change in the acuity of the “number sense”: the approximate number system in 3-, 4-, 5-, and 6-year-olds and adults. *Dev. Psychol.* 44 1457–1465 10.1037/a0012682 18793076

[B18] Halberda J. Ly R. Wilmer J. B. Naiman D. Q. Germine L. (2012). Number sense across the lifespan as revealed by a massive Internet-based sample. *Proc. Natl. Acad. Sci. U.S.A.* 109 11116–11120 10.1073/pnas.1200196109 22733748 PMC3396479

[B19] Halberda J. Mazzocco M. M. M. Feigenson L. (2008). Individual differences in non-verbal number acuity correlate with maths achievement. *Nature* 455 665–668 10.1038/nature07246 18776888

[B20] Howell D. C. (1997). *Statistical Methods for Psychology*. Belmont CA: Duxbury Press

[B21] Inglis M. Attridge N. Batchelor S. Gilmore C. (2011). Non-verbal number acuity correlates with symbolic mathematics achievement: but only in children. *Psychon. Bull. Rev.* 18 1222–1229 10.3758/s13423-011-0154-1 21898191

[B22] King-Smith P. E. Grigsby S. S. Vingrys A. J. Benes S. C. Supowit A. (1994). Efficient and unbiased modifications of the QUEST threshold method: theory, simulations, experimental evaluation and practical implementation. *Vision Res.* 34 885–912 10.1016/0042-6989(94)90039-6 8160402

[B23] Libertus M. E. Brannon E. M. (2010). Stable individual differences in number discrimination in infancy. *Dev. Sci.* 13 900–906 10.1111/j.1467-7687.2009.00948.x 20977560 PMC2966022

[B24] Libertus M. E. Feigenson L. Halberda J. (2011). Preschool acuity of the approximate number system correlates with school math ability. *Dev. Sci.* 14 1292–1300 10.1111/j.1467-7687.2011.01080.x 22010889 PMC3338171

[B25] Libertus M. E. Odic D. Halberda J. (2012). Intuitive sense of number correlates with math scores on college-entrance examination. *Acta Psychol.* 141 373–379 10.1016/j.actpsy.2012.09.009 PMC3495271 23098904

[B26] Lindskog M. Winman A. Juslin P. Poom L. (2013a). Measuring acuity of the approximate number system reliably and validly: the evaluation of an adaptive test procedure. *Front. Psychol.* 4:510 10.3389/fpsyg.2013.00510 PMC3734355 23964256

[B27] Lindskog M. Winman A. Juslin P. (2013b). Are there rapid feedback effects on Approximate Number System acuity? *Front. Hum. Neurosci.* 7:270 10.3389/fnhum.2013.00270 PMC3679493 23781191

[B28] Lipkus I. M. Samsa G. Rimer B. K. (2001). General performance on a numeracy scale among highly educated samples. *Med. Decis. Making* 21 37–44 10.1177/0272989X0102100105 11206945

[B29] Mazzocco M. M. M. Feigenson L. Halberda J. (2011a). Impaired acuity of the approximate number system underlies mathematical learning disability (dyscalculia). *Child Dev.* 82 1224–1237 10.1111/j.1467-8624.2011.01608.x 21679173 PMC4411632

[B30] Mazzocco M. M. M. Halberda J. Feigenson L. M. (2011b). Preschoolers’ precision of the approximate number system predicts later school mathematics performance. *PLoS ONE* 6:e23749 10.1371/journal.pone.0023749 PMC3173357 21935362

[B31] Nieder A. Freedman D. J. Miller E. K. (2002). Representation of the quantity of visual items in the primate prefrontal cortex. *Science* 297 1708–1711 10.1126/science.1072493 12215649

[B32] Noël M.-P. Rousselle L. (2011). Developmental changes in the profiles of dyscalculia: an explanation based on a double exact-and-approximate number representation model. *Front. Hum. Neurosci.* 5:165 10.3389/fnhum.2011.00165 PMC3243900 22203797

[B33] Nys J. Ventura P. Fernandes T. Querido L. Leybaert J. Content A. (2013). Does math education modify the approximate number system? A comparison of schooled and unschooled adults. *Trends Neurosci. Educ.* 2 13–22 10.1016/j.tine.2013.01.001

[B34] Park J. Brannon E. M. (2013). Training the approximate number system improves math proficiency. *Psychol. Sci.* 24 2013–2019 10.1177/0956797613482944 23921769 PMC3797151

[B35] Peters E. Vastfjall D. Slovic P. Mertz C. K. Mazzocco K. Dickert S. (2006). Numeracy and decision making. *Psychol. Sci.* 17 407–413 10.1111/j.1467-9280.2006.01720.x 16683928

[B36] Piazza M. Facoetti A. Trussardi A. N. Berteletti I. Conte S. Lucangeli D. (2010). Developmental trajectory of number acuity reveals a severe impairment in developmental dyscalculia. *Cognition* 116 33–41 10.1016/j.cognition.2010.03.012 20381023

[B37] Piazza M. Izard V. Pinel P. Le Bihan D. Dehaene S. (2004). Tuning curves for approximate numerosity in the human intraparietal sulcus. *Neuron* 44 547–555 10.1016/j.neuron.2004.10.014 15504333

[B38] Piazza M. Pica P. Izard V. Spelke E. S. Dehaene S. (2013). Education enhances the acuity of the nonverbal Approximate Number System. *Psychol. Sci.* 24 1037–1043 10.1177/0956797612464057 23625879 PMC4648254

[B39] Pica P. Lemer C. Izard V. Dehaene S. (2004). Exact and approximate arithmetic in an amazonian indigene group. *Science* 306 499–503 10.1126/science.1102085 15486303

[B40] Price G. R. Palmer D. Battista C. Ansari D. (2012). Nonsymbolic numerical magnitude comparison: reliability and validity of different task variants and outcome measures, and their relationship to arithmetic achievement in adults. *Acta Psychol.* 140 50–57 10.1016/j.actpsy.2012.02.008 22445770

[B41] Raven J. Raven J. C. Court J. H. (1998). *Manual for Raven’s Progressive Matrices and Vocabulary Scales. Section 4, The Advanced Progressive Matrices*. Oxford: Oxford Psychologists Press

[B42] Reyna V. F. Nelson W. L. Han P. K. Dieckmann N. F. (2009). How numeracy influences risk comprehension and medical decision making. *Psychol. Bull.* 135 943–973 10.1037/a0017327 19883143 PMC2844786

[B43] Stanovich K. E. West R. F. (1998). Individual differences in framing and conjunction effects. *Think. Reason.* 4 289–317 10.1080/135467898394094

[B44] Starr A. Libertus M. E. Brannon E. M. (2013). Number sense in infancy predicts mathematical abilities in childhood. *Proc. Natl. Acad. Sci.* 110 18116–18120 10.1073/pnas.1302751110 24145427 PMC3831472

[B45] Stoianov I. Zorzi M. (2012). Emergence of a “visual number sense” in hierarchical generative models. *Nat. Neurosci.* 15 194–196 10.1038/nn.2996 22231428

[B46] Stoianov I. Zorzi M Umiltà C. (2004). The role of semantic and symbolic representations in arithmetic processing: insights from simulated dyscalculia in a connectionist model. *Cortex* 40 194–196 10.1016/S0010-9452(08)70948-1 15174483

[B47] Szûcs D. Nobes A. Devine A. Gabriel F. C. Gebuis T. (2013). Visual stimulus parameters seriously compromise the measurement of approximate number system acuity and comparative effects between adults and children. *Front. Psychol.* 4:444 10.3389/fpsyg.2013.00444 PMC3715731 23882245

[B48] Tokita M. Ishiguchi A. (2012). Behavioral evidence for format-dependent processes in approximate numerosity representation. *Psychon. Bull. Rev.* 19 285–293 10.3758/s13423-011-0206-6 22231727

[B49] Verguts T. Fias W. (2004). Representation of number in animals and humans: a neural model. *J. Cogn. Neurosci.* 16 1493–1504 10.1162/0898929042568497 15601514

[B50] Zebian S. Ansari D. (2011). Differences between literates and illiterates on symbolic but not nonsymbolic numerical magnitude processing. *Psychon. Bull. Rev.* 19 93–100 10.3758/s13423-011-0175-9 22033982

